# How will the Covid-19 pandemic shape the future of primary care undergraduate teaching? Understanding modifications and developments deployed by UK academic units of primary care, and their implications for the future

**DOI:** 10.1186/s12909-023-04710-6

**Published:** 2023-10-10

**Authors:** Michael Harrison, Lauren Hall, Hugh Alberti

**Affiliations:** School of Medical Education, Faculty of Medical Sciences, Newcastle University, Newcastle upon Tyne, UK

**Keywords:** Covid-19, Primary care, Undergraduate, Clinical Placement

## Abstract

**Background:**

Primary care has been under-represented in its contribution to the academic literature base on Covid-19 developments. We sought to understand how teaching and learning was modified and developed by primary care academic leaders to support the continuation of primary care-orientated learning during the Covid-19 pandemic; and explore how these changes may shape future educational delivery in primary care.

**Methods:**

We adopted a qualitative approach, using semi-structured interviews of seven General Practice Heads of Teaching (GP HoTs) from UK medical schools. We used mixed deductive and inductive coding to analyse interview transcripts. Modifications and developments were coded to four a priori themes (clinical off-site; clinical on-site; synchronous remote; asynchronous remote). We concurrently used inductive coding to identify developments that did not readily fit into these categories. To understand how participants perceived the developments may shape primary care teaching in the future, we carried out an inductive thematic analysis.

**Results:**

A range of modifications and developments were described. Examples of developments include: GP practices being provided with increased flexibility to support ongoing provision of clinical placements (*on-site clinical*), examples of initiatives enabling students to consult remotely from their homes (*off-site clinical*), transfer of face-to-face teaching to remote formats (*synchronous remote*) and development of new, interactive on-line teaching materials (*asynchronous remote*). One additional theme arose inductively: collaboration and co-operation.

For future implications, five themes arose: the evolution of flexible and hybrid clinical placement models; an increased role for telemedicine; increased networking and collaboration; increased active student involvement in patient care; and opportunities for community-based teaching afforded by the pandemic.

**Conclusion:**

This study highlights how teaching was modified to support the continuation of primary care-based learning during the Covid-19 pandemic, and implications for the future. Collaboration and placement flexibility were notable features in the response. Participants perceived that flexible placement models containing a mixture of clinical on-site with remote synchronous and asynchronous teaching and learning activities, may persist into the post-Covid era. Further research is required to understand which developments become routinely embedded into primary care teaching in the post-Covid era and explain how and why this occurs.

**Supplementary Information:**

The online version contains supplementary material available at 10.1186/s12909-023-04710-6.

## Introduction

The Covid-19 pandemic has caused significant disruption to undergraduate medical education, and a wealth of literature has emerged describing responses made in response to Covid-19 by undergraduate educators. The need to maintain physical distancing resulted in the rapid deployment of remote synchronous and asynchronous learning, often through moving education from classrooms to virtual spaces [1–4]. Ongoing workplace-based learning has been facilitated through numerous adaptations, including telemedicine and students seeing patients face to face with mitigated risk [1–4].

However, it has been recognised that primary care has been under-represented in its contribution to this literature base, and recommendations have been made for increased scholarly output from primary care settings [1]. Since the conception of this project, further literature from UK primary care undergraduate teaching settings has been published with respect to telemedicine training [5–9], blended placement models [10–12] and use of recorded GP consultations as learning tools [13]. There are several notable examples of UK-based studies. Darnton et al. [6] piloted 35 medical students consulting from home remotely with patients. They found their educational initiative to be acceptable and educationally valuable, and cited numerous advantages of consulting in this way, including reduced travel and dead time for students. Noonan et al. [11] developed a hybrid model of placement in response to the Covid-19 pandemic, utilising a mixture of virtual tutorials and clinical placement time. They described how their hybrid teaching model has proven to be a resilient and superior teaching model and has facilitated recruitment of a wider range of GPs who would usually not be involved in teaching. They also described numerous benefits to using authentic videoed patient cases during virtual tutorials. Dow et al. [13] also described use of pre-recorded consultations as a teaching tool for year one students, finding it to be reproducible, time-efficient, and beneficial to students. Finally, Patel and Taggart [10] found an ongoing high quality of delivery of medical education, with no attrition in student experience, when using virtual small group teaching to replace small group learning in primary care.

This study was developed in response to calls for increased scholarly output from primary care. Whilst individual evaluations of undergraduate, primary care-based teaching developments have been published, no literature exists, to the best of the authors’ knowledge, describing the approaches taken by primary care leaders to support the continuation of primary care teaching and learning in response to the Covid-19 pandemic. There is also a lack of understanding of how these changes may shape primary care teaching provision in the future. The latter is particularly important as much of the academic interest now concerns with trying to understand which of the multitude of described educational initiatives is ‘effective, desirable and sustainable’ moving into a post-Covid era [4].

In the UK, General Practice (GP) Heads of Teachers (HOTs) are academic leads responsible for overseeing the strategic delivery of academic activity relating to university-based primary care teaching [14]. The GP HoTs group has representation from all UK medical schools and provides a national forum for exchange of information in relation to undergraduate primary care education [14]. The group works closely with the Society of Academic Primary Care (SAPC) and Royal College of General Practitioners (RCGP) to provide a unified approach to the provision of primary care undergraduate education in the UK. Therefore, GP HoTs offer credible insight into the changes made to undergraduate teaching in primary care in response to the Covid-19 pandemic, and how this may impact on the future of primary care teaching.

Thus, the aim of this study was to provide an overview of developments and modifications deployed by UK academic units of primary care in response to Covid-19; and then ask the question of ‘what next?’ with respect to these changes. To do this, we asked the following research questions:

1. How was teaching and learning modified and developed by primary care academic leaders to support the continuation of workplace-based, and non-workplace-based, primary care-orientated learning during the Covid-19 pandemic?
2. How to GP HoTs perceive these changes may shape educational delivery in primary care in the post-pandemic era?

## Materials and methods

### Theoretical stance

In this study, we aimed to understand the real-world implications of Covid-19, though exploring the actions, experience and views of key academic leaders within UK primary care teaching. Because of this, we decided to adopt a pragmatic approach. Epistemologically, pragmatism is premised on the idea that research should focus on practical understandings of concrete, real-world issues [15]. Pragmatism recognises that research should emanate from a desire to produce useful and actionable knowledge and the interconnectedness of experience, knowing and acting [16].

### Sample and data collection

An invitation to participate in this study was included as an additional question in a survey being completed by GP HoTs for a separate research project [17]. The study described here has not been completed in collaboration with any other research project or team and does not form part of any larger project. Seven GP HoTs expressed interest in being involved in this study and were e-mailed directly by lead researcher (MH) to arrange individual interviews. Interviews were conducted remotely by MH via Microsoft Teams between February and June 2021, using a semi-structured interview guide. Written consent was obtained from participants.

**Fig. 1:**
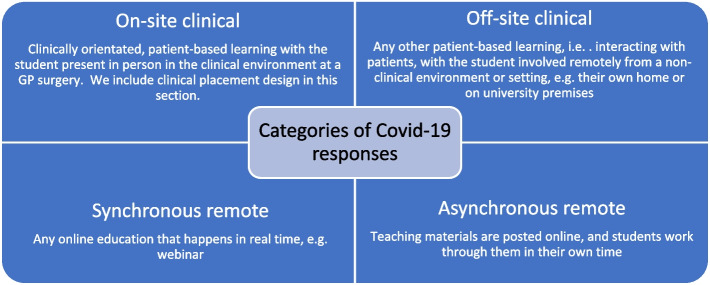
Categories of Covid-19 responses

Initial questions about developments and modifications were guided by four categories of Covid-19 responses (clinical on-site, clinical off-site, synchronous remote, asynchronous remote; see Fig. 1 for definitions). These four categories had initially been devised for use in a UK national survey on student teaching and learning during the Covid-19 pandemic [18] and are used with consent by the lead author. These categories were chosen for use in this study as the study team felt they provided a contemporaneous overview of Covid-19 responses within medical education, which could then be applied to a primary care context. Hence, the categories were used to provide a starting point to understand Covid-19 responses deployed within primary care The interview schedule (available to view as an additional Appendix) also allowed us to identify developments that did not readily fit into the four a priori categories, and to explore how the changes may shape future educational delivery. Interviews were audio-recorded and transcribed verbatim. All identifiers were removed to ensure participant confidentiality.

### Data analysis

 NVivo was used to document the analysis. There were two stages to analysis. The two parts of analysis were performed sequentially, as follows:

1. A mixed deductive and inductive analysis was performed to understand types of modifications and developments. These were coded deductively to one or more of the four a priori themes. There was concurrent use of inductive coding to identify developments that did not readily fit into these categories. Any new categories arising from inductive coding were iteratively reviewed and revised as further transcripts were read and coded, eventually producing a final representation of modifications and developments.
2. To explore perceptions of how these changes may shape primary care teaching in the future, an inductive thematic analysis was performed: following initial familiarisation with the data, codes were generated based on significant or recurrent utterances. Repeated reading of transcripts allowed iterative analysis with constant comparison of codes both within and between transcripts. Codes were subsequently grouped into potential themes with further review and refinement producing a final thematic map.

LH completed initial coding of all the transcripts and then helped develop, in conjunction with MH, the additional inductive themes for RQ1, and all the themes for RQ2. There was then iterative review of the transcripts by both MH and LH to review and refine the themes. Discrepancies in theme emergence were resolved through team discussion and further iterative review of the transcripts. Rigour was also maintained through documentation of the theme emergence, prolonged engagement with the transcripts, and acknowledgement of our own position in the research process (‘reflexivity’). MH and HA are academic GPs with an interest in community-based teaching and learning, whereas LH is a junior doctor who is developing an interest in GP teaching. We acknowledge that our own thoughts and biases have the potential to impact on the research process, but took active steps to mitigate this risk, including use of ‘bracketing’. We believe that this ensures the results faithfully reflect the data.

## Results

### Interview participants

Seven interviews were conducted, representing approximately a quarter of all GP HoTs. Five were from English medical schools and two were from Scottish medical schools. Three were male and four were female.

### Developments and modifications

**Fig. 2:**
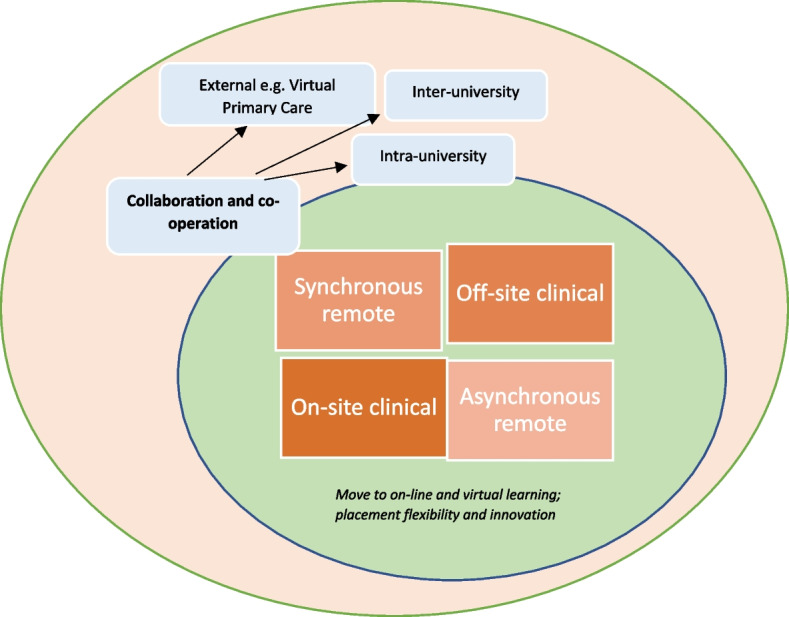
Modifications and developments deployed by academic units of primary care

A range of developments and modifications were reported. One further theme arose inductively from the data: collaboration and co-operation. Figure 2 provides a thematic overview of the developments and modifications.

The following description provides a narrative account of the four core, and one additional, categories. Note that, whilst each is discussed discreetly, a degree of overlap between categories inevitably exists, and this is discussed where appropriate. Table 1 provides specific examples for each of the four core categories.

**Table 1:** Examples of modifications and developments from the core categories

**On-site clinical**	**Off-site clinical**
Placement flexibility for practices and GP tutors Remote consulting: telephone and video Altered learning opportunities, e.g. audit work, helping with vaccinations	Opportunities for students to consult with patients from home remotely
**Synchronous remote**	**Asynchronous remote**
Early years communication skills teaching changed to a synchronous remote format Large group teaching changed to a synchronous remote format Small group teaching/de-brief time changed from in-person to remote format	Development of new, interactive on-line teaching resources Use of videoed patient encounters, e.g. Virtual Primary Care Recorded lectures on-line

#### On-site clinical

Participants described advocating the use of a more flexible approach to GP placement at their respective institutions. This was used as a way of supporting GP practices to continue to take students on clinical placement despite clinical pressures and staff absences. The flexible approach included less prescription about the amount of time students were expected to be at the practice experiencing direct patient contact, and the day(s) of the week students were expected to attend. A range of other learning opportunities were often provided as a replacement for time that would usually be spent with direct patient contact. Some of these activities were done with students on-site in the practice, e.g., quality improvement activities, whilst others were organised locally by practices, e.g., helping at a Covid-19 vaccine hub. Other learning opportunities were sometimes provided directly by the university and consisted of a range of synchronous and asynchronous remote learning activities to supplement direct patient contact.

We said to them (GP teachers): to reduce the burden on the three days in practice we would expect them to only spend half a day on each of those days in consultation type experience and the other half could be done on almost anything else, so we encouraged them to do more quality improvement stuff, more audits, anything to help really (Participant 1).

Telemedicine, usually using remote consulting with video or telephone with the student present at the practice, was suggested to support ongoing patient contact. Most participants reported developing guidance and teaching materials relating to telemedicine, although usually this did not include specific recommendations to practices regarding which type of telemedicine students should use. This decision was mostly left up to individual GP tutors and their practices, who could choose how to allow students to interact remotely with patients based on their own individual clinical working practises and preferences.

Practices are getting students to do some remote consulting and some video type consulting. We’ve said to the GP’s the students should be doing what you’re doing in your day-to-day practice, we don’t want the GPs to try and create things that aren’t happening in practice, we want them to have an authentic learning experience of what clinical practice is (Participant 3).

There was a general perception that, although the more flexible clinical placement structure resulted in some loss of standardisation of student experience, there were numerous unintended benefits of this approach. This included enhanced GP teacher recruitment in some areas, an increased range and authenticity of student experience, increased student understanding of pandemic working, and increased student opportunities to be directly involved in patient care and practice activities.

#### Off-site clinical

There were a few examples reported by participants of GP practices enabling students to consult remotely from their own homes, using a variety of formats and with differing levels of tutor involvement. However, the examples described were pre-dominantly local and ad hoc, and there was an absence of examples of formal initiative development to facilitate remote consulting in the institutions of those interviewed. Remote consulting with patients in this way was sometimes used as a replacement for face-to-face consulting for early years clinical visits. Concerns around confidentiality and information governance, alongside the perception that other initiatives developed may be of more value educationally, appeared to be the biggest barriers to implementation.

We generally didn’t have students speaking to patients from their homes…..some medical schools did….but I think the reason we didn’t push ahead with that is because we very rapidly developed other resources.. so we never ended up going down the remote consulting route, except with some of the early years placements (Participant 5).

#### Synchronous remote

There were a wide variety of examples of teaching and learning activities that were changed to a synchronous remote format, in response to the pandemic. A variety of different technologies were used, including Microsoft Teams, Zoom and Noodle.

Almost all early-years campus- based teaching organised and delivered by academic units of primary care was changed to a synchronous remote format. This included large and small group teaching. Many units reported successfully changing their early years communication skills teaching with actors to a virtual synchronous format.

Many units also changed some small group teaching on clinical placement to a synchronous remote format. This included changing group-based discussion and patient de-briefs from a face to face to a remote format. There was one example of virtual one-to-one tutorials between GP mentors and students being organised as part of a pastoral support when clinical placements were cancelled at the start of the pandemic.

Our communication skills teaching went totally online, So, we basically lifted the format and put it online and it seemed to work really well (Participant 3).

#### Asynchronous remote

Participants described the rapid development of on-line resources in response to the pandemic. This involved development of the university’s pre-existing virtual learning environment e.g. Blackboard. Resources were in a wide range of forms, including recorded lectures and new on-line learning modules. Participants commented that these resources often replaced pre-existing, and sometimes outdated, paper resources with more interactive, on-line resources.

Participants commented that synchronous remote formats were often used in conjunction with asynchronous formats, such as remote small group tutorials. This ‘flipped classroom’ format was felt to be most effective, as it offered opportunities for discussion and feedback. One notable example that was frequently cited was the use of Virtual Primary Care (VPC) [19], which provided students with access to real life consults often used as an asynchronous activity, followed up with group discussion as a synchronous activity.

Instead of a classroom-based face to face session, we delivered it remotely with flipped classroom approach with work given to them at the beginning of the work they could work on (Participant 4).

### Collaboration and co-operation

Collaboration and co-operation emerged inductively as an important theme that facilitated continuation of primary care teaching. We felt it was sufficiently heterogenous to be included as a separate theme. Collaboration and co-operation occurred on numerous levels, as described below.

#### Intra-university collaboration

There were numerous examples of intra-university collaboration. Several participants described increased levels of engagement between community teachers and academic faculty staff through increased use of technology, such as WhatsApp, to provide regular updates and briefings. There was a general perception that this had strengthened pre-existing teacher networks. One participant described clusters of GP practices working collaboratively, including the provision of shared tutorials delivered remotely; and discussed the potential for Primary Care Networks (PCNs), to facilitate community-based teaching opportunities, e.g., through vaccine hubs. Another participant discussed improved teamworking and co-operation with secondary care colleagues responsible for undergraduate medical education delivery within their institution.

We’ve worked really, really hard to engage with GP tutors. I think this level of engagement will continue… and methods of engagement, like through WhatsApp groups and more frequent briefing of tutors and keeping in touch with others…I think that will be a good thing for the future. (Participant 2).

#### Inter-university collaboration

Numerous participants described enhanced collaboration between the different academic units of primary care, and that this had been facilitated via the SAPC GP HoTs group. They reported that the group allowed for sharing of ideas, resources, and support. Collaboration was seen as a way of providing solutions to shared problems experienced by different institutions. For example, one participant described how a lack of good quality video resources led to the creation of shared learning resources and external collaboration with Virtual Primary Care (VPC). Inter-university collaboration also occurred via other avenues, such as the Scottish Universities Network and regional GP HoTs meetings. There was a perception that this kind of collaborative working had increased in response to the pandemic.

I think the GP Head of Teaching group has been really useful. I think it’s been a great support with sharing of ideas and sharing resources…we also locally have our own meetings in between the national GP HoTs meetings (Participant 7).

### External collaboration

The principal example of external collaboration cited by participants was VPC. VPC was developed in conjunction with the Medical Schools Council (MSC) and the TV Producers of Channel 5’s GP: Behind Closed Doors, and provides access to 150 real life GP consultations [19]. Many participants described using the VPC resource in a variety of teaching formats at their institution.

We had a need for video material and that was when we got the idea of approaching ‘GP’s behind closed doors’….and that was very fruitful and we got their interest very quickly and they said ‘yes we’ve got thousands of consultations, we are happy to work with you and make the videos into a form that could be used for learning through an online platform’ (Participant 6).

### How will the developments shape primary care teaching in the future?

**Fig. 3:**
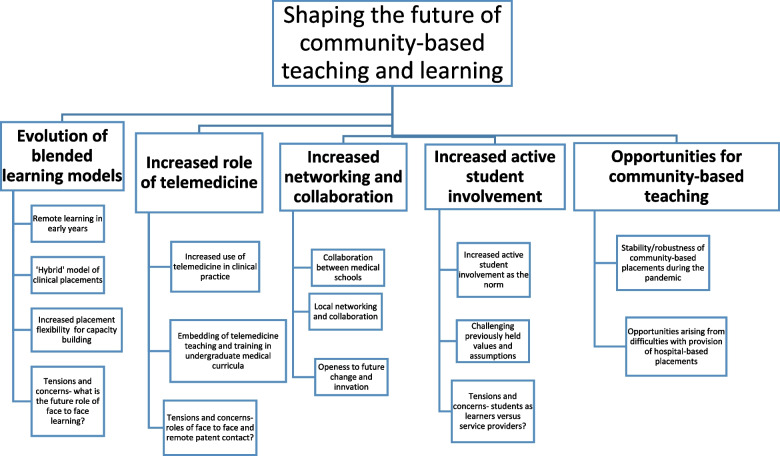
How will the developments shape primary care teaching in the future?

Five themes emerged in this section, as demonstrated in Fig. 3. Each theme is discussed separately, with illustrative quotes provided. It is noted that a diverse range of views were expressed by participants. The following narrative aims to capture both the commonalities, and differences and uncertainties, within the study participants’ views.

#### Evolution of blended or hybrid learning models

A common theme arising from participants’ responses was the likely embedding of blended or hybrid learning models, particularly with respect to clinical placements. Whilst recognising that in-person presence on clinical placement remained essential, there was a general view that a mixture of learning methods would remain. It was envisaged that this may include a mixture of synchronous remote activities, e.g., small group webinars, and asynchronous remote learning activities, in addition to the usual face-to-face presence on clinical sites. Some participants described successful integration of hybrid learning models within their undergraduate medical course as a permanent change to their curriculum. Others reported blended learning models being a feasible option for early years clinical contact for the future.

What we’ve carried on and emerged to now is a hybrid model where we’ve managed to get our GP tutors set up to use the Virtual Primary Care. (Participant 4).

The general impression was that a mixture of these different activities may be desirable from a pedagogical perspective because they offer opportunities for self-study using on-line teaching resources, group discussion, feedback, and a wider variety of teaching methods. However, it was also acknowledged that there may be a range of drivers for hybrid model implementation, including advantages to students, e.g. reduced travel time, financial considerations, and placement capacity. There was a general impression that more flexible clinical placements would likely remain, e.g. less specification for GP teachers regarding exact placement activities. Some participants expressed views that this could help GP teacher recruitment and retention.

I think there will be more openness to other solutions than ‘students must spend their entire time in placement’ the whole time. I think there will be an openness to being more flexible in models. I’m not convinced we’ll go back to students spending hours and hours on placement. (Participant 3).

There were, however, differences of opinion between participants regarding the value of hybrid placements, with some feeling that the inherent value of learning in person on placement is irreplaceable. Several participants were keen for clinical placements to return to similar formats to pre-pandemic, with most time spent in person on placement. These participants reflected on the significant benefits of learning in person on placement, such as opportunities to integrate with the clinical team and practise clinical skills. One participant was concerned that future decisions to deliver hybrid models may be driven pre-dominantly by teacher recruitment challenges rather than pedagogical value.

It will be very interesting to see whether then hybrid models are possibly used to manage shortage of placements rather than purely for pedagogical point of view. (Participant 5).

#### Increased role of telemedicine

Many participants felt that the trend towards increased telemedicine use in clinical practice meant that it was essential that teaching on telemedicine and remote consulting was embedded into undergraduate medical curricula. Telemedicine competence was felt to be a key skill needed for future doctors. Integration of telemedicine teaching and training was already occurring at many of the institutions, with some describing the development of vertical strands of telemedicine teaching spanning all course years.

We recognise that remote consultations are going to continue and so we are developing a vertical learning stream of remote consultations throughout all five years in a kind of spiral curriculum way (Participant 4).

However, there were a range of views, sometimes conflicting, about the role and value of telemedicine within primary care clinical placements. Whilst some viewed telemedicine solely as a complement, but not a substitute, for face-to-face contact, others noted the inherent value of telemedicine in student learning, such as in the development of history taking skills. One participant commented that, despite students experiencing fewer patient contacts, they felt that each patient contact carried a higher level of educational value. Current trends within primary care clinical practice towards reverting to face-to-face appointments on the back of the pandemic, alongside concerns that students may not be getting enough face-to-face patient contact on placement, created further tension about the role of telemedicine. There was no single unifying consensus on the future role, and educational value, of telemedicine during primary care clinical placements.

I still think there is the feeling that it’s not the same doing it by phone and what do you learn from doing it? I think actually you can learn a lot about history taking and nuances of language from doing it remotely. (Participant 7).

#### Increased collaboration and networking

Some participants discussed the potential for innovative collaborative working between clinical care providers to support future clinical placements, such as between GP practices and PCNs. One participant discussed the continuation of clustered groups of three or four practices with shared virtual group tutorials, that had been initially initiated during the pandemic. Another participant discussed the idea of using a PCN as a hub site as a way of potentially recruiting satellite practices. Some of the participants reflected on changes to clinical working practices during the pandemic, such as improved inter-practice working triggered by Covid-19 vaccine hubs; and how this may influence undergraduate medical education delivery in the future. The use of technology, particularly virtual tutorials, was often envisaged as a way of facilitating interaction between geographically different groups of students on placement.

One of the things that has happened with vaccination hubs is that PCN’s are starting to work together a lot better. I think there is a spirit of working together as a PCN that has been engendered by the Covid vaccinations really. It is an area that we are currently looking at as being hopefully useful for recruitment. (Participant 2).

Participants felt that that the increased collaboration and sharing of ideas between GP HoTs that occurred in response to the pandemic was a positive development. They were keen for this collaborative spirit to continue moving into the future. There was a general perception that leaders within medical schools and primary care were more open to change and creative because of working through a pandemic. Ongoing improved communication between academic staff and GP practices was also viewed as important moving out of the pandemic.

#### Increased active student involvement on placement

Many participants commented that the requirement for increase placement flexibility meant that students had had more opportunities to become actively involved in patient care and within clinical teams. It was noted that some students had become involved in additional paid or unpaid or voluntary activities during the pandemic. One participant reflected that students may have been ‘held back’ before the pandemic. There was one mention of students being given essential worker status during the pandemic. The potential for more active involvement in patient care and clinical teams was seen as a positive development, and often cited as something that they would like to continue in the future. It was perceived that this was of benefit educationally and valued by students. Some commented that they had been particularly reassured by student feedback, which had been comparable to pre-pandemic feedback. However, some concern remained about how to balance this more flexible approach, which facilitates more active involvement in care, with equity of student experience and adequate coverage of curriculum learning outcomes.

I’m hoping that in the future students will be much more involved in helping out. I’ve talked to the students a lot about what they’ve learnt through that….even though they are not perhaps on track with the formal curriculum (Participant 2).

#### Opportunities for community-based learning

Many participants felt that, apart from initial cessation of clinical placement at the start of the pandemic, primary care placements had been robust and maintained a high quality of student teaching throughout the pandemic. Some participants discussed some of the challenges hospitals had faced in maintaining clinical placement provision during the pandemic, often due to clinical and staffing pressures. One participant discussed how his primary care unit had been given opportunities to deliver more placement time within GP because hospitals were struggling to accommodate students. It was felt that primary care had been given opportunities to showcase its potential as a provider of high-quality medical education and offered optimism for the delivery of more teaching in a community-based setting in the future.

This year, because they (hospital rotations) didn’t want students…it was an opportunity for us to say ‘well we can take students for two weeks rather than one’ (Participant 1).

## Discussion

In this study, we have provided an overview of developments and modifications deployed by a sample of UK academic units of primary care in response to Covid-19, and gathered expert opinion on how this may shape educational delivery in primary care in the future.

### Comparison with pre-existing literature

Our results provide a broad understanding of modifications in relation to both workplace-based, and non-workplace-based, learning. In relation to non-workplace-based learning, our findings generally mirror those by Stojan et al. [4], who described the rapid transition of existing teaching to online formats using synchronous and asynchronous formats. Notable examples from our findings include the successful transfer of communication skills teaching to a synchronous remote format and use of video consults as a synchronous or asynchronous learning activity. Stojan et al. [4] discussed the potential advantages of using synchronous and asynchronous formats together, which may encourage ‘both virtual engagement and interactivity, while providing opportunities for more flexible, self-directed learning’. Our study supports the idea that there are co-benefits of using synchronous and asynchronous learning activities together, and that this combination may find the ‘sweet spot’ between autonomous self-study and opportunities for group discussion, reflection, and feedback.

Grafton-Clarke et al. [3] identified that on-line learning was used as an adaptation to facilitate workplace- based learning during the pandemic, although most of the literature identified in their systematic review originated from the hospital context. Our findings confirm that this adaptation also occurred in the UK primary care context. Our study also provides an overview of the strategic approach taken to support the continuation of clinical placements during the pandemic. One of the key modifications made by GP HoTs was adopting a flexible approach to primary care placement to support teaching practices. Our study suggests that this may have had an unintended consequence of affording students the opportunity to take increasingly active and legitimate roles in the primary healthcare team. The educational value of legitimate participation in clinical practice is well documented. Lave and Wenger’s well known theory of legitimate peripheral participation conceptualised learning as a situated activity in which students build relationships with other members in informal networks and groupings in a workplace [20]. These Communities of Practice (CoP) play an important role in enriching students’ learning as they move towards full participation through active participation [20].

The value of situated learning provides a juxtaposition between the ‘traditional’ model of primary care clinical placement, in which students spend most of their time on placement in-person, and hybrid or blended models of clinical placement, in which students spend more time interacting virtually. Research has highlighted that creating a sense of belonging and community is harder in the virtual world [4] and it remains to be seen whether much of the tacit and unintended learning that occurs on placement can occur as effectively in a virtual format. However, there may be numerous benefits of hybrid models, for example reduced travel time for students and increased teacher retention [11], that may be desirable for university leaders and other stakeholders. The latter benefit may be particularly attractive because of GP teacher shortages [17]. GP teaching expansion is occurring in the context of national and international calls to deliver more undergraduate teaching in a community setting [21, 22]. Early evaluative data is emerging regarding the use of hybrid models in the post-Covid era. Zoonan et al. [11] describe successful integration of virtual tutorials into the curriculum of a UK medical school, utilising a mixture of synchronous and asynchronous activities alongside clinical placement time. They report pedagogical benefits of using a hybrid placement design and describe it being well received by students and teachers alike.

Whilst remote consultations will likely play an important role in the delivery of primary care clinical services moving forward [23, 24], the future role of telemedicine within undergraduate primary care teaching is less clear. GP leaders in our study expressed a range of views on this subject. This uncertainty is reflected in the literature base. Students have expressed reservations that remote consulting brings fewer opportunities to examine patients and can narrow the patient case-mix [25]. However, Darnton et al. [7] suggested that remote consults may be essential in ensuring an adequate case mix for students, may help develop certain skills, and provide additional time to reflect on cases. Whilst Darnton et al. [7] suggested that students felt as much as part of the team on GP placement during Covid-19, Al-Bedaery et al. [8] suggested that students felt isolated and less part of the clinical team when consulting remotely.

Collaboration was an important part of the response to the pandemic by academic units of primary care. Collaboration between institutions facilitated sharing of ideas and mutual support. External collaboration with a TV company facilitated the production of VPC. Stojan et al. [4] observed few multi-institutional collaborations in undergraduate medical education compared to postgraduate medical education during the Covid-19 pandemic. Our findings provide an excellent example of multi-institutional collaboration that provided a solution to a shared problem, and which has fuelled enthusiasm for future collaborative working.

### Strengths and limitations

This study utilised interviews of GP HoTs, who provide credible insights into the developments deployed at their respective institutions and offer expert opinion. The use of interviews, and a mixed deductive inductive analysis, allowed us to adopt an open-minded approach to understand innovative Covid-19 developments, whilst ensuring our results were framed within the current understanding of Covid-19 responses from the literature base.

This study interviewed around a quarter of all GP HoTs. It is likely that our results do not capture all modifications and developments deployed by GP HoTs. The results of the second research question attempts to provide a broad synthesis of responses, whilst acknowledging a multiplicity of opinions, some of which were contrasting. We accept that there will be some opinions not captured by our results. As such, it would be incorrect to assume that the results accurately describe the strategic approach adopted at every academic unit of primary care, or that the study reflects the opinion of every individual GP HoT.

By collecting qualitative, and not quantitative data, we did not manage to collect data about the frequency of deployment of the various developments across all institutions. Our data also did not include developments and modifications in relation to assessment or faculty development.

### Implications for future practice and research

Our findings suggest that flexible placement models, which include a mixture of clinical on-site and remote synchronous and asynchronous activities, may persist into the post-Covid era. These hybrid or blended placement models may aid the recruitment and retention of GP teachers and have wider intended and unintended benefits. There is a need to develop an understanding of the benefits and dis-benefits of such models when deployed in a ‘non-emergency’ context. Thus, we recommend longitudinal evaluation of programmes of study in institutions who continue to implement such models.

There is a need for further research to clarify how best to integrate remote consulting into GP placements. Current evidence is conflicting and, given that remote consulting is likely to persist in primary care clinical practice, there is an urgent need for clarification on this matter. There is also a need for evaluation of student teaching programmes on telemedicine delivered by primary care staff.

One of the commendable responses by GP HoTs was the collaborative effort. Collaboration between different institutions helped provide mutual support and shared solutions to problems faced by academic leaders. VPC provides an excellent example of external collaboration. We recommend that this collaborative spirit continues in the post-pandemic era, and that further notable examples of collaboration be evaluated, so learning for the wider educational community can be extracted from such initiatives.

## Conclusion

The Covid-19 pandemic resulted in an explosion of published literature of educational interventions deployed in response to the pandemic. However, primary care has been under-represented in the literature base. This study helps improve our understanding of Covid-19 responses within primary care. In our study, whilst many of the developments could be mapped to pre-existing themes, we found that collaboration and placement flexibility were important features in the response.

As medical schools adapt to the cessation of Covid-19 restrictions, many institutions face uncertainty as to which educational developments to keep in their curriculum. There is also a need to understand how and why successful and unsuccessful implementation of educational developments occur. We have shed some light on this subject, by gaining expert opinion on how primary care teaching may be shaped by these developments. The experts interviewed in our study perceived that community-based clinical placements may become more flexible, including a combination of on-line and remote learning with more traditional on-site clinical activities, as part of a so-called ‘hybrid’ or ‘blended’ model. The study also identifies areas that require further consideration and research. This includes uncertainty about the future role of telemedicine within undergraduate curricula, the value of situated learning on clinical placement and the extent to which teacher recruitment challenges and placement capacity will act as drivers to change rather than the pedagogical value of educational interventions.

### Supplementary Information

**Additional file 1: Appendix 1.** GP HOTS Project interview schedule.

## Data Availability

The datasets used and/or analysed during the current study are available from the corresponding author on reasonable request.

## Abbreviations

GP HoTs: GP Heads of Teaching
VPC: Virtual Primary Care
SAPC: Society of Academic Primary Care
