# Supplementary material for: How will the Covid-19 pandemic shape the future of primary care undergraduate teaching? Understanding modifications and developments deployed by UK academic units of primary care, and their implications for the future
Source: BMC Med Educ. 2023 Oct 10;23:746. doi: 10.1186/s12909-023-04710-6 (PMC10566052; doi:10.1186/s12909-023-04710-6)
Supplement: Supplementary file 1 — Additional file 1: Appendix 1. GP HOTS Project interview schedule. [file 12909_2023_4710_MOESM1_ESM.docx]

# Appendix 1: GP HOTS Project interview schedule

## Introduction

*Many thanks for your time. You should have received an information leaflet and also signed a consent form. Do you have any questions about the project prior to commencing? The interview will be recorded but information in the interview will remain anonymous.*

*As I’m sure you are aware, the Covid-19 pandemic has significantly affected under-graduate teaching and learning within medical schools. We are keen to learn more about the changes that have occurred to teaching within primary care and, importantly, how this may affect community-based teaching in the future.*

## Questions

What types of modifications and innovations have been developed by your departments of primary care in response to Covid-19?

- How does this fit with wider modifications made within the medical school?
- If requiring prompting, interviewees can be asked to think of modifications and innovations in terms of the four domains of learning as follows:
  - Clinical on-site
  - Clinical off-site
  - Synchronous remote
  - Asynchronous remote

We believe that changes to medical education brought about as a result of Covid-19 could permanently affect medical education in primary care in the future

- What do you think are the medium- and long-term impact of these changes on teaching within primary care, and why?
- Which of the changes/innovations do you suspect will continue to be used in primary care teaching post-Covid-19, and why?
- Which of the changes/innovations do you feel will place a lesser role in primary care teaching once the Covid-19 pandemic is over, and why?
- Which of the changes/innovations do you believe can result in sustainable improvements to primary care teaching, and why? (Improvements could be in relation to a wide variety of aspects, including teaching quality, teaching capacity, assessment, etc.)
- How do you feel that these changes relate to the longer- term plans for community-based teaching within the UK? E.g., expansion pf primary care teaching, promotion of GP as a career choice, details of the Wass report etc.
- How will implementation and embedding of any changes be facilitated?

## Finish

Many thanks for your participation in this project? Do you have any questions before we finish? Are you still happy to proceed with your involvement in the project?
